# ADHD-like behaviors caused by inactivation of a transcription factor controlling the balance of inhibitory and excitatory neuron development in the mouse anterior brainstem

**DOI:** 10.1038/s41398-020-01033-8

**Published:** 2020-10-21

**Authors:** Francesca Morello, Vootele Voikar, Pihla Parkkinen, Anne Panhelainen, Marko Rosenholm, Aki Makkonen, Tomi Rantamäki, Petteri Piepponen, Teemu Aitta-aho, Juha Partanen

**Affiliations:** 1 Molecular and Integrative Biosciences Research Programme, Faculty of Biological and Environmental Sciences, University of Helsinki, P.O. Box 56, 00014-University of Helsinki, Helsinki, Finland; 2 Neuroscience Center, University of Helsinki, P.O. Box 29, 00014-University of Helsinki, Helsinki, Finland; 3 Department of Pharmacology, University of Helsinki, P.O. Box 63, 00014-University of Helsinki, Helsinki, Finland; 4 Institute of Biotechnology, University of Helsinki, P.O. Box 56, 00014-University of Helsinki, Helsinki, Finland; 5 Drug Research Program, Division of Pharmacology and Pharmacotherapeutics, Faculty of Pharmacy, University of Helsinki, P.O. Box 56, 00014-University of Helsinki, Helsinki, Finland; 6 SleepWell Research Program, Faculty of Medicine, University of Helsinki, Helsinki, Finland

**Keywords:** ADHD, Molecular neuroscience

## Abstract

The neural circuits regulating motivation and movement include midbrain dopaminergic neurons and associated inhibitory GABAergic and excitatory glutamatergic neurons in the anterior brainstem. Differentiation of specific subtypes of GABAergic and glutamatergic neurons in the mouse embryonic brainstem is controlled by a transcription factor *Tal1*. This study characterizes the behavioral and neurochemical changes caused by the absence of *Tal1* function. The *Tal1*^*cko*^ mutant mice are hyperactive, impulsive, hypersensitive to reward, have learning deficits and a habituation defect in a novel environment. Only minor changes in their dopaminergic system were detected. Amphetamine induced striatal dopamine release and amphetamine induced place preference were normal in *Tal1*^*cko*^ mice. Increased dopamine signaling failed to stimulate the locomotor activity of the *Tal1*^*cko*^ mice, but instead alleviated their hyperactivity. Altogether, the *Tal1*^*cko*^ mice recapitulate many features of the attention and hyperactivity disorders, suggesting a role for *Tal1* regulated developmental pathways and neural structures in the control of motivation and movement.

## Introduction

Brain functions behind movement, motivated behavior, attention, impulse control, and learning are regulated by neurons in the anterior brainstem. Extensive research has shown the roles of dopaminergic neurons in the substantia nigra pars compacta (SNpc) and in the ventral tegmental area (VTA) in these behaviors^1,2^. Altered dopaminergic neurotransmission has also been implicated as one of the mechanisms of hyperactivity disorders, including attention-deficit hyperactivity disorder (ADHD)^2^. Furthermore, the symptoms of ADHD are alleviated by drugs that evoke dopamine release, such as amphetamine.

Dopamine neurons receive both excitatory and inhibitory input from diverse brain regions. These regulatory neurons include GABAergic and glutamatergic neurons in the dopaminergic nuclei or in their vicinity, in particular in the substantia nigra pars reticulata (SNpr), ventral tegmental area (VTA), rostromedial tegmental nucleus (RMTg), and laterodorsal tegmental nucleus (LDTg)^3,4^. Importantly, the inhibitory GABAergic and excitatory glutamatergic neurons in these nuclei not only regulate the dopaminergic neurons but also project to other brain structures. For example, the VTA GABAergic neuron projections to the prefrontal cortex (PFC) and the nucleus accumbens (NAc) are important for arousal and assessment of reward^5,6^, the SNpr GABAergic neuron projections to the superior colliculus and the thalamus provide the main output of the basal ganglia network regulating voluntary movement^7^, and the RMTg regulates both the dopaminergic and serotonergic systems, which have also been implicated in regulation of impulsivity^8^.

Most of the GABAergic neurons in the midbrain dopaminergic nuclei share developmental origins and regulatory mechanisms, which differ from the rest of the midbrain GABAergic neurons^9,10^. These neurons are born in a specific domain of the ventral rhombomere 1 of the hindbrain, where their differentiation depends on a transcription factor Tal1^10^. In this region of the brain, Tal1 functions as a cell fate selector gene promoting GABAergic differentiation at the expense of the alternative glutamatergic neuron identities^10^. In conditional *Tal1* mutant mice, where the *Tal1* gene is inactivated in the midbrain and rhombomere 1 (*En1*^*Cre*^; *Tal1*^*flox/flox*^; *Tal1*^*cko*^), all GABAergic neurons in the RMTg, VTA and most of the neurons in the posterior part of the SNpr fail to develop. In contrast to the posterior SNpr, the anterior SNpr, not derived from the rhombomere 1, as well as other midbrain GABAergic neurons are intact in the *Tal1*^*cko*^ mice. In addition to the GABAergic neurons associated with the dopaminergic nuclei in the ventral midbrain, the *Tal1*^*cko*^ mice lack specific brainstem GABAergic neuron subgroups, including the neurons of the ventral tegmental nucleus (VTg)^11^^,12^. Interestingly, the VTg was recently implicated in regulation of diencephalic medial mammillary bodies and the memory function in rats^13,14^. On the contrary to GABAergic neurons, in the *Tal1*^*cko*^ mice, the number of glutamatergic neurons is increased in the interpeduncular nucleus and the LDTg, which provides excitatory projections to the VTA^10,15^.

As the brainstem nuclei housing Tal1 dependent neurons have been implicated in the control of the dopamine neurons and in the regulation of movement, motivated behavior and learning, we studied how the altered development of the *Tal1*^*cko*^ mice affects behavior and the function of the dopaminergic system. In these mice, we observed many behavioral features resembling clinical ADHD, including hyperactivity, increased motor impulsivity, altered response to reward, and impaired learning. Interestingly, the *Tal1*^*cko*^ mice had no marked changes in their dopaminergic system but, similar to the ADHD patients, showed a paradoxical calming response to pharmacologically stimulated dopamine release.

## Materials and methods

Methods are described briefly below, see Supplementary Information for detailed descriptions.

### Mice

Mice carrying *En1*^*Cre*^^16^ and *Tal1*^*flox*^^11^ alleles were crossed to generate *En1Cre/+*; *Tal1flox/flox* (*Tal1*^*cko*^) mice. In the *Tal1*^*cko*^ mice, the *En1Cre* allele drives recombination tissue-specifically both in the midbrain and rhombomere 1, but this results in a failure of brainstem GABAergic neurogenesis only in the embryonic rhombomere 1^10,17,18^. Four cohorts of wild-type (total *n* = 74, females; *n* = 56, males) and *Tal1*^*cko*^ (total *n* = 48 females; *n* = 38, males) mice were generated in outbred ICR background for behavioral analyses. The littermates of the *Tal1*^*cko*^ mice, carrying different combinations of the *Tal1flox* and *En1Cre* alleles, were included in the wild-type group. The mice were analyzed at the age of 3–4 months. The body weight of the *Tal1*^*cko*^ mice is lower than that of wild-type mice (males, WT, 35.7 g, *Tal*^*cko*^, 27.4 g; females, WT, 27.0 g, *Tal1*^*cko*^, 21.1 g). Behavioral testing was approved by the National Animal Experiment Board of Finland (License numbers ESAVI/7548/04.10.07/2013, ESAVI/8132/04.10.07/2017) according to the EU legislation (Directive 2010/63/EU) harmonized with Finnish legislation.

### Drugs

The following drugs were used: amphetamine (GlaxoSmithKline), GBR12909 dihydrochloride (Tocris), atomoxetine (Orion Pharma, capsule contents dissolved in saline followed by brief centrifugation), SCH23390 hydrochloride (Sigma), and raclopride tartrate (Sigma) were dissolved in sterile saline. All drug solutions were injected at the volume of 10 ml/kg.

### Behavioral analyses

Following behavioral analyses were performed: Open field, Elevated zero maze, Light-dark box, Social approach, Novel object recognition, 3-compartment test for sociability, Rotarod, Multiple static rods, Hot-plate, Pre-pulse inhibition, Forced swim test, Fear conditioning, T-maze, Water maze, Circadian activity, Nest construction, Burrowing, Marble burying test, Grooming, Stress-induced hyperthermia, IntelliCage for flexible sequencing task, motor impulsivity, saccharin preference and delay discounting. For the order of the behavioral tests and used cohorts of animals, see Supplementary Information.

Amphetamine-conditioned place preference was used to assess the rewarding properties of amphetamine in wild-type and *Tal1*^*cko*^ mice in an unbiased and counterbalanced manner as described^19^ with modifications^20^.

### Immunohistochemistry and DA neurons counts

To analyze DA neuron number, the mouse brains were processed as previously described^10^.

### Measurement of tissue dopamine by HPLC

For brain monoamine measurements the samples were obtained from the relevant dissected brain regions using a mouse brain matrix as previously described^21^. In addition, prefrontal cortex (PFC) was collected from in front of the striatal slice after removal of the olfactory bulbs. Dopamine and its metabolites were analyzed as described previously^22^ using HPLC with electrochemical detection.

### Measurement of dopamine release by cyclic voltammetry

Acute striatal mouse brain slices were prepared, dopamine elicited from axon terminals, and transient dopamine signals (release and uptake) were detected using fast-scan cyclic voltammetry as previously described^21^. Amphetamine (5 µM, d-amphetamine hemi-sulfate, Sigma-Aldrich, St. Louis, MO) was added to the recording solution after a stable baseline of stimulated dopamine transients was reached, and the responses were recorded until the amphetamine-induced dopamine efflux through dopamine transporter was measured.

### In vivo microdialysis of dopamine release

Mice were surgically implanted with indwelling guide cannuli targeted into the NAc or dorsal striatum. After a recovery period, amphetamine was injected at 3 mg/kg, i.p., and thereafter dialysate was collected for dopamine measurement by HPLC with electrochemical detection^21^.

### Data analysis and statistics

The sample size was based on earlier experience on behavioral and other testing procedures. The experimental animals were subjected to the experimental groups in a random manner. Randomization was applied for the testing order in conventional tests (Random number calculators available at https://www.graphpad.com/quickcalcs/randMenu/). Blinding was applied at all steps during testing and analysis—experimenter was unaware of genotypes and treatments, the group codes were opened after collecting the data and running the analysis. Exclusion criteria for behavioral data were either sickness of the animal, verified measurement error, or technical failure. No data were excluded. Behavioral data were evaluated using an ANOVA model with genotype (wild-type, *Tal1*^*cko*^ mice) and sex as between subject factors. Within subject factors were added as needed when exploring the dependence of genotype effects on place or time (e.g., open field activity, water maze, IntelliCage etc.). Significant interactions and where necessary significant main effects were further explored by post-hoc tests or by splitting the ANOVA model, as appropriate. The data are presented as mean +/− standard error of the mean, and n indicates the number of biological replicates. Data between sexes were pooled for figures unless otherwise stated.

## Results

Development of anterior brainstem GABAergic and glutamatergic neurons, involved in the regulation of dopaminergic pathways and in the basal ganglia output, is altered in the *Tal1*^*cko*^ mice^10^. Therefore, we analyzed basal ganglia regulated behaviors in these animals. The summary of the behavioral analyses is presented in Table 1 (Supplementary Material).

### The *Tal1*^*cko*^ mice display a distinct pattern of hyperactivity and motor impulsivity

**Fig. 1:**
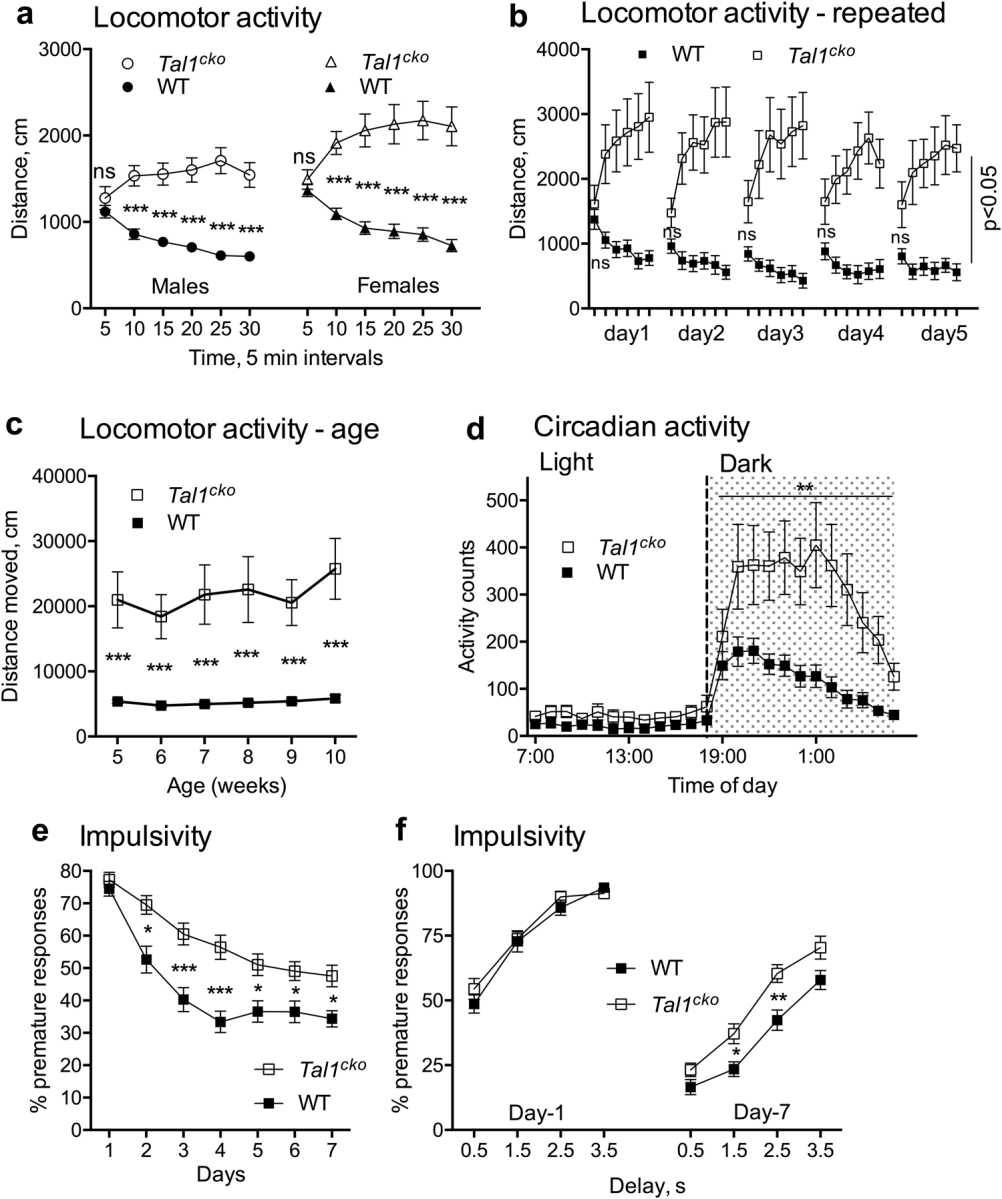
Hyperactivity and motor impulsivity in *Tal1*^*cko*^ mice. **a** In the open field test, the locomotor activity of the *Tal1*^*cko*^ mice was normal in the beginning of the trial, but in contrast to normal within-trial habituation and decrease of activity in wild-type mice, the *Tal1*^*cko*^ mice showed progressively increasing activity (RM ANOVA genotype × time interaction F5,850 = 57.7, *p* < 0.0001), leading to increased locomotor activity (distance traveled in 30 min) in *Tal1*^*cko*^ mice (RM ANOVA genotype F1,170 = 107.5, *p* < 0.0001). *N* = 174 (54 WT M; 42 KO M; 43 WT F; 35 KO F). **b** Open field test repeated on 5 consecutive days showed persistent hyperactivity (RM ANOVA genotype F1,20 = 11.1, *p* = 0.0033) and no between-trial habituation of female *Tal1*^*cko*^ mice (RM ANOVA days F4,40 = 1.3, *p* = 0.28). First timebin on all days, no genotype difference *p* > 0.05, other timebins *p* < 0.05, Bonferroni’s test. *N* = 23 (12 KO; 11 WT). **c** Longitudinal open field testing showed hyperactivity in *Tal1*^*cko*^ mice from juvenile age through adulthood (RM ANOVA genotype F1,30 = 78.44, *p* < 0.001. *N* = 32 (6 KO F; 26 WT F). **d** Analysis of circadian activity in single housed mice showed higher activity of the *Tal1*^*cko*^ mice in home cage setting during the dark cycle (RM ANOVA genotype F1,90 = 11.3, *p* = 0.0011). **p < 0.01, between genotypes, ANOVA. *N* = 104 (WT M 40; KO M 31; WT F 11; KO F 12). **e** Impulsivity as measured by suppression of motor action in the IntelliCage. The *Tal1*^*cko*^ mice displayed more premature responses and inability to wait at longer delays along with delayed learning (RM ANOVA genotype F1,22 = 18.4, *p* = 0.0003, interaction genotype × days F6,132 = 3.9, *p* = 0.0012). *N* = 24 (13 WT F; 11 KO F). **f** Percentage premature responses at increasing delays on day 1 shows no difference between the genotypes (RM ANOVA genotype F1,22 = 0.523, *p* = 0.477). At day 7, the *Tal1*^*cko*^ mice are impaired at delays of 1.5 s and 2.5 s in the impulsivity testing (RM ANOVA genotype F1,22 = 10.59, *p* = 0.0036). *p < 0.05, **p < 0.01, ***p < 0.001, between genotypes, Bonferroni’s test. *N* = 24 (13 WT F; 11 KO F).

To analyze activity and movement, we first recorded *Tal1*^*cko*^ mice for both within-trial and between-trial locomotor activity in the open field test. In the beginning of the trial, *Tal1*^*cko*^ mice had normal locomotor activity (Fig. 1a) but as wild-type mice decreased their activity over the course of the trial, the *Tal1*^*cko*^ mice failed to habituate to the test environment and instead displayed within-trial hyperactive behavior (Fig. 1a). Repeated open field testing further revealed a between-trial deficiency in habituation (Fig. 1b). In a longitudinal testing of the activity, *Tal1*^*cko*^ mice were hyperactive from juvenile age until adulthood (Fig. 1c). Consistent with the open field test, individually housed *Tal1*^*cko*^ mice showed increased activity during long term monitoring in the home cage (Fig. 1d). However, and as seen in wild-type mice, their peak activity occurred in the dark cycle while the activity during the inactive light cycle remained unchanged suggesting no obvious change in the circadian rhythm (Fig. 1d). We tested impulsive behavior by training animals to suppress motor action (nosepoke) in the IntelliCage in order to get access to drinking water and found that *Tal1*^*cko*^ mice made more premature nosepokes, which means that mice reacted before the intended time elapsed and suggests a failure to wait. Both groups improved over time, but *Tal1*^*cko*^ mice performed worse throughout the experiment (Fig. 1e, f). Motor coordination of the *Tal1*^*cko*^ mice was slightly better in the rotarod test (Fig S1a) while a subtle deficit appeared in the multiple rod test (Fig S1b). This difference might be explained by different demands of the tasks, as in the rotarod test the hyperactive phenotype may rescue the slight deficit in coordination.

### Changes in reward behavior in the *Tal1*^*cko*^ mice

**Fig. 2:**
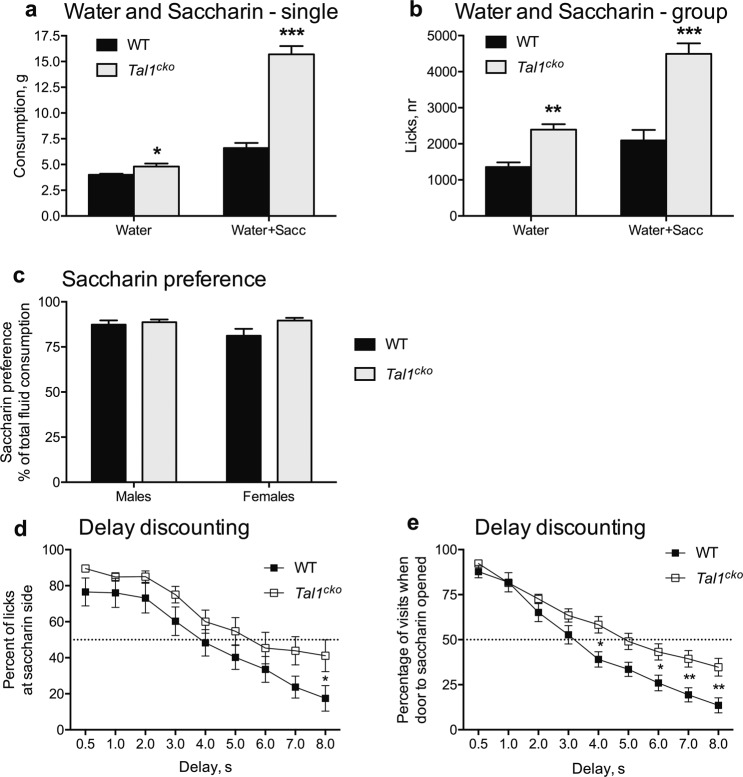
Reward behavior. **a**. Liquid consumption in single housed male mice The *Tal1*^*cko*^ mice showed increased drinking when only water was available and the difference was even larger when saccharin was available (ANOVA genotype F1,45 = 94.6, *p* < 0.001; ANOVA condition F1,45 = 314.0, *p* < 0.001; interaction F1,45 = 117.2, *p* < 0.001; *p < 0.05, ***p < 0.001, between genotypes, *t*-test) *N* = 47 (29 WT M; 18 KO M). **b**. Liquid consumption (lick number) of female mice, housed in social groups in the IntelliCage, was increased in the *Tal1*^*cko*^ mice (F1,77 = 26.7, *p* < 0.0001 with water only; F1,77 = 27.5, *p* < 0.0001 with water and saccharin), *N* = 79 (43 WT F; 36 KO F). **c** Preference for sweet taste (0.5% saccharin), as calculated from total consumption of liquid, was not different between the *Tal1*^*cko*^ and wild-type mice in either sex (F1,77 = 0.002, *p* = 0.96, *p* < 0.05) (F1,77 = 0.002, *p* = 0.96, p < 0.05). **d** Delay discounting in the IntelliCage—preference for saccharin expressed as percentage of licks at increasing delays to opening the door to saccharin side (RM ANOVA genotype F1,22 = 3.0, *p* = 0.097). In the wild-type mice, saccharin preference was significantly lower than 50% already at a delay of 6 s (*p* = 0.04, one sample *t*-test), whereas it remained close to 50% in the *Tal1*^*cko*^ mice mice even at 8 s delay (*p* = 0.33, one-sample *t*-test). The difference was also detected between the genotypes (*p* = 0.047, *t*-test) at 8 s delay, *p < 0.05, between genotypes, *N* = 24 (13 WT F; 11 KO F). **e** Delay discounting—percentage of visits with door to saccharin opened at increasing delays (RM ANOVA genotype F1,22 = 6.4, *p* = 0.019), N = 24 (13 WT F; 11 KO F).

Next, we asked if reward processing and motivated behavior was altered in the *Tal1*^*cko*^ mice. Water consumption of the *Tal1*^*cko*^ mice was slightly increased at baseline (Fig. 2a). When given a choice between plain water and a palatable solution containing 0.5% saccharin in water, both the wild-type and the *Tal1*^*cko*^ mice strongly preferred the saccharin solution (Fig. 2c), but the availability of saccharin provoked the *Tal1*^*cko*^ mice to consume considerably greater amounts of the saccharin containing solution. This suggests that sensing reward is enhanced in the *Tal1*^*cko*^ mice. To further study the reward behavior, we analyzed the response of female mice to saccharin in the Intellicage system. Similar to the males, the availability of saccharin strongly increased drinking of the female *Tal1*^*cko*^ mice (Fig. 2b). When the access to saccharin was delayed, in comparison to immediate access to water, the *Tal1*^*cko*^ mice showed reduced delay discounting as measured by enhanced saccharin preference (Fig. 2d), and persistence in waiting to get access to saccharin (Fig. 2e) at longer delays compared to the wild-type mice.

### No changes in stress and anxiety-like behavior in the *Tal1*^*cko*^ mice

**Fig. 3:**
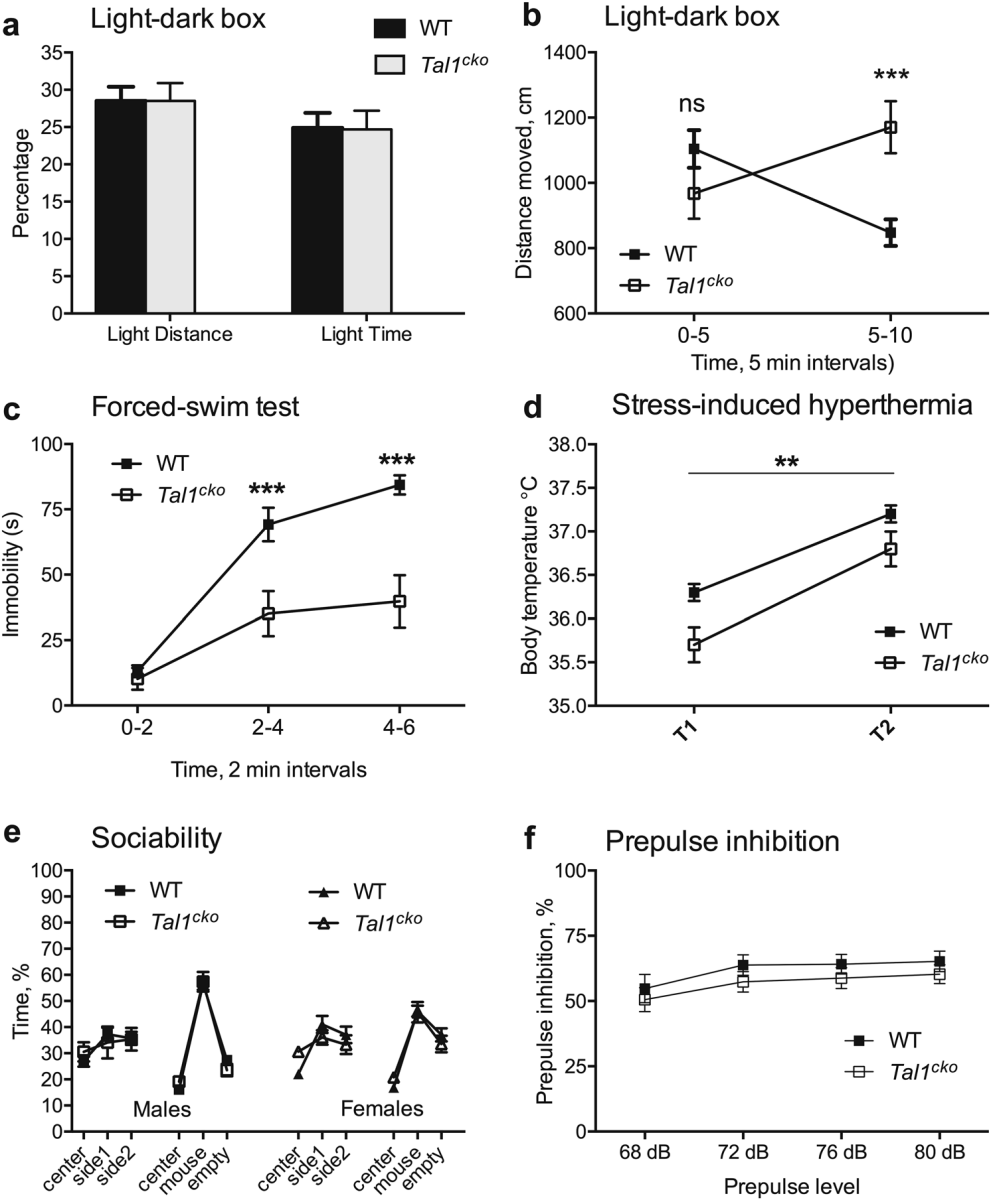
Anxiety and mood. **a** The Light-dark box test revealed no difference between the wild-type and the *Tal1*^*cko*^ mice in anxiety-like behavior. Both groups displayed similar avoidance of brightly illuminated compartment as shown by the percentage of time and distance moved in light compartment (time, F1,84 = 0.002, *p* = 0.96; distance, F1,84 = 0.0004, *p* = 0.98), *N* = 88 (27 M WT; 19 M KO; 24 F WT; 18 F KO). **b** Activity of the *Tal1*^*cko*^ mice (total distance traveled) increased significantly during the second half of the testing period in the light-dark box (RM ANOVA genotype x time interaction F1,84 = 53.2, *p* < 0.0001). ***p < 0.001, between genotypes, Bonferroni’s test, *N* = 88 (27 M WT; 19 M KO; 24 F WT; 18 F KO). **c** In the forced swim test, the time of immobility in 2 min intervals was significantly reduced in the *Tal1*^*cko*^ mice (RM ANOVA genotype F1,28 = 39.3, *p* < 0.0001, interaction genotype x time F2,56 = 29.1, *p* < 0.0001), ***p < 0.001, between genotypes, Bonferroni’s test, *N* = 30 (18 M WT; 12 M KO). **d** Stress-induced hyperthermia. The *Tal1*^*cko*^ mice had lower basal body temperature (F1,22 = 8.5, *p* = 0.0081), and stress induced equal hyperthermic effect in both *Tal1*^*cko*^ and wild-type mice (F1,22 = 0.5, *p* = 0.49). **p < 0.01, between genotypes, ANOVA. *N* = 24 (14 M WT; 10 M KO). **e** Sociability test. Time with the stimulus mouse was not altered between the genotypes (F1,32 = 0.007, *p* = 0.94). *N* = 36 (9 M WT; 7 M KO; 13 F WT; 7 F KO). **f** Prepulse inhibition of acoustic startle reflex was not different between the groups (RM ANOVA genotype F1,28 = 1.4, *p* = 0.25), but increased with the increasing sound pressure level of pre-pulse (RM ANOVA pre-pulse level F3,84 = 9.3, *p* < 0.001; genotype x pre-pulse level interaction F3,84 = 0.2, *p* = 0.9), *N* = 30 (18 M WT; 12 M KO).

As the anterior brainstem contains nuclei regulating the behavioral state associated with stress and anxiety, we tested the *Tal1*^*cko*^ mice for changes in anxiety behavior. In the light-dark box, the proportion of distance moved and time spent in the light compartment was similar between the wild-type and the *Tal1*^*cko*^ mice, suggesting no change in the levels of anxiety (Fig. 3a), a hypothesis also supported by normal behavior in the elevated zero-maze test (Table 1, Supplementary Material). As in the open-field test, at the beginning of the light-dark test the *Tal1*^*cko*^ mice had normal locomotor activity, which later developed into hyperactivity (Fig. 3b). We found similar outcome in the forced swim test (Fig. 3c), a test widely used to assess despair behavior, in which the *Tal1*^*cko*^ mice were more active after displaying normal activity in the beginning of the test. This appears consistent with the increased saccharin consumption (see above) suggesting reduced depressive behavior. However, it is also possible that the reduced immobility of *Tal1*^*cko*^ mice is simply caused by their hyperactivity. We observed unaltered behavior of the *Tal1*^*cko*^ mice in tests of stress-induced hyperthermia and social behavior (Fig. 3d, e). In addition, we found prepulse inhibition, a translatable measure of sensorimotor gating, to be unaltered in the *Tal1*^*cko*^ mice (Fig. 3f).

### Defects of learning and complex behaviors in the *Tal1*^*cko*^ mice

**Fig. 4:**
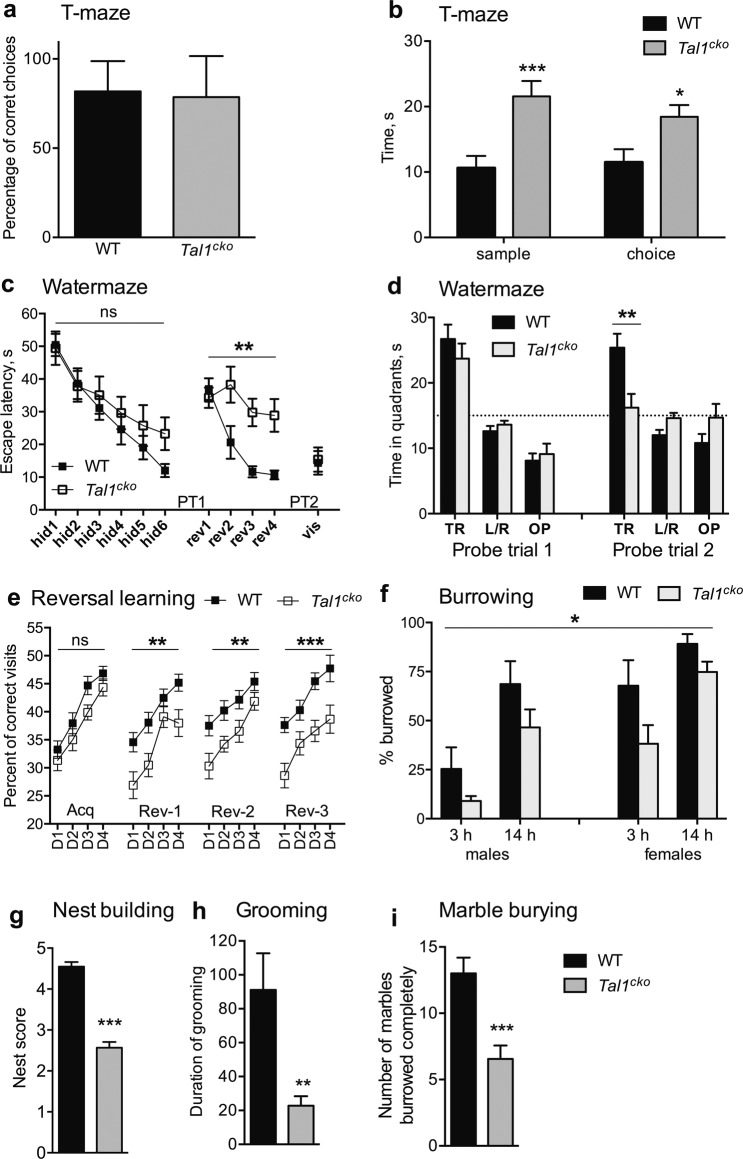
Cognitive functions, learning and memory, and innate behaviors. **a** Spontaneous alternation (working memory) in T-maze test was not altered in the *Tal1*^*cko*^ mice (ANOVA genotype F1,32 = 0.32, *p* = 0.58), *N* = 36 (9 M WT; 7 M KO; 13 F WT; 7 F KO). **b** Time for completing the trials in T-maze was longer in the *Tal1*^*cko*^ mice (RM ANOVA F1,32 = 12.6, *p* = 0.0012), *N* = 36 (9 M WT; 7 M KO; 13 F WT; 7 F KO). **c** Spatial learning (latency to find the platform) in water maze-test. There was no difference between the wild-type and *Tal1*^*cko*^ mice in learning to find escape platform in acquisition phase (RM ANOVA genotype F1,20 = 1.0, *p* = 0.33, trial block F5,100 = 19.9, *p* < 0.0001, no interaction between genotype and trial blocks). However, the *Tal1*^*cko*^ mice were unable to improve their performance in reversal learning when platform was moved to the opposite quadrant (RM ANOVA genotype F1,20 = 14.2, *p* = 0.0012, trial block F3,60 = 9.6, *p* < 0.0001, interaction genotype x trial block F3,60 = 4.5, *p* = 0.0064). Navigation to visible platform was not different between the groups, *N* = 22 (12 M WT; 10 M KO). **d** Search bias (preference to quadrant) in probe trials without escape platform available confirmed no difference between the groups after initial acquisition (in probe trial 1 where both groups spent similar amount of time in trained quadrant (F1,20 = 0.9, *p* = 0.35). However, in probe trial 2, after reversal learning sessions, the *Tal1*^*cko*^ mice did not show any preference neither to new nor old quadrant, and spent significantly less time in correct quadrant as compared to the wild-type (F1,20 = 9.2, *p* = 0.066). PT = probe trial. *N* = 22 (12 M WT; 10 M KO). **e**. Flexible sequencing task in IntelliCage. The *Tal1*^*cko*^ mice acquired the task in the first phase with no difference to wild-type mice (RM ANOVA genotype F1,42 = 3.1, *p* = 0.09). However, learning of the *Tal1*^*cko*^ mice remained significantly worse in each of the following reversal phases, suggesting reduced behavioral flexibility (RM ANOVA genotype F1,42 > 8.8, *p* > 0.005), *N* = 44 (24 F WT; 20 F KO). **f** Burrowing was reduced in *Tal1*^*cko*^ mice (RM ANOVA genotype F1,36 = 7.0, *p* = 0.0118), *N* = 40 (10 M WT; 10 M KO; 10 F WT; 10 F KO). **g** Nest building was impaired in the *Tal1*^*cko*^ mice (F1,112 = 111.2, *p* < 0.0001), *N* = 116 (40 M WT; 31 M KO; 23 F WT; 22 F KO). **h** *Tal1*^*cko*^ mice engaged significantly less time in grooming during 10 min test (F1,20 = 9.4, *p* = 0.0061), N = 22 (11 F WT; 11 F KO). **i** Marble burying test. The *Tal1*^*cko*^ mice buried less marbles than wild-type group (F1,31 = 19.1, *p* = 0.0001), *p < 0.05, **p < 0.01, ***p < 0.001, between genotypes, *t*-test, Bonferroni’s test, or ANOVA, *N* = 35 (6 M WT; 7 M KO; 11 F WT; 11 F KO).

Next, we analyzed learning and complex species-specific behavior of the *Tal1*^*cko*^ mice. In the T-maze test for working memory, the spontaneous alternation of the *Tal1*^*cko*^ mice was not affected (Fig. 4a). However, it took longer for the *Tal1*^*cko*^ mice to complete the trial (Fig. 4b).

In the Morris water maze test, the *Tal1*^*cko*^ mice were able to learn to find the hidden platform (Fig. 4c). The *Tal1*^*cko*^ mice also showed preference to the trained quadrant in the following probe trial (Probe trial 1, Fig. 4d). However, when the platform was moved to the opposite quadrant, the *Tal1*^*cko*^ mice were severely impaired in learning the new location (Fig. 4c) and showed a completely random search strategy after training (Probe trial 2, Fig. 4c, d). This finding of reduced behavioral flexibility was further corroborated by impaired reversal learning of the *Tal1*^*cko*^ mice in the Intellicage flexible sequencing task (Fig. 4e).

In the novel object recognition test, the *Tal1*^*cko*^ mice showed again the inverse pattern of locomotor habituation, but this did not significantly change their relative preference for the novel object (Fig S2a, b). Interestingly, contextual fear conditioning was impaired in *Tal1*^*cko*^ mice (Fig S2c).

We then analyzed mouse species-specific behaviors, such as grooming, nest construction, burrowing and marble burying, likely requiring animals to focus and concentrate their efforts on specific tasks. In the *Tal1*^*cko*^ mice, all of these behaviors were significantly reduced (Fig. 4f–i).

### Dopaminergic system in the *Tal1*^*cko*^ mice

**Fig. 5:**
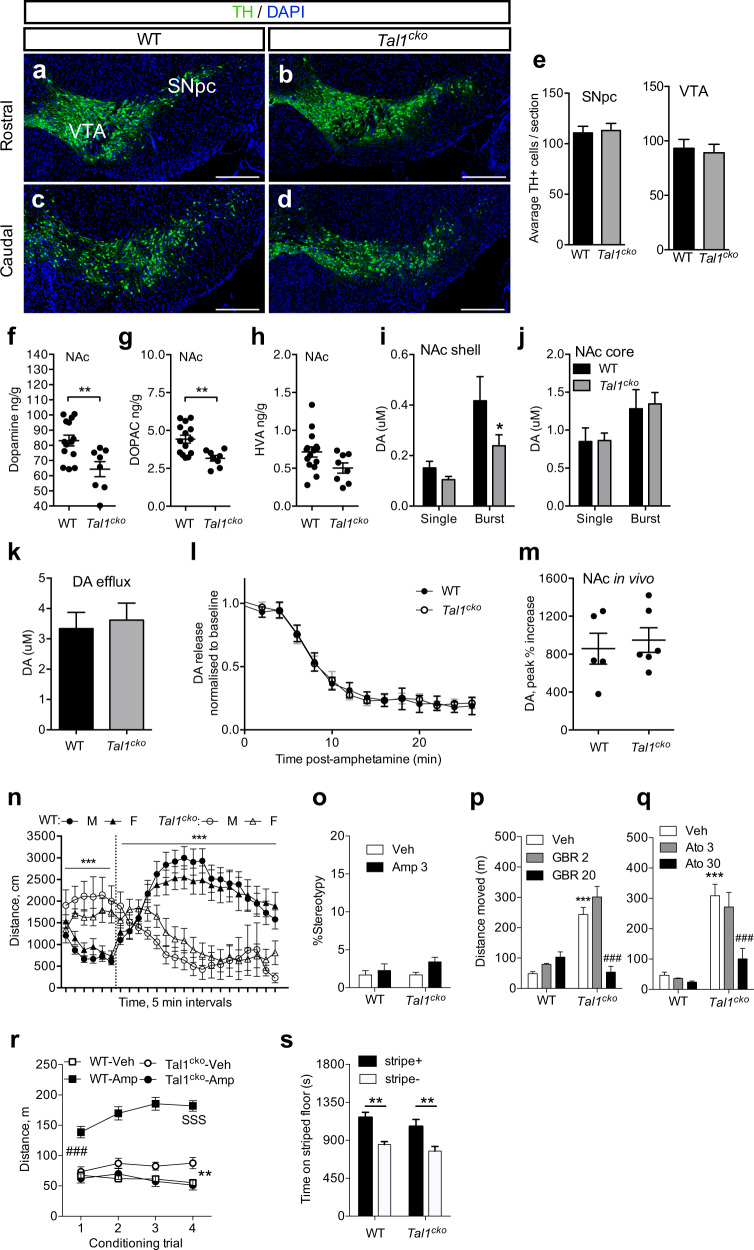
Dopaminergic system in *Tal1*^*cko*^ mice. **a**–**d** Immunohistochemical analysis of midbrain TH+ neurons in the wild-type and *Tal1*^*cko*^ mice. Representative images of TH-labeled coronal sections through the VTA and the SNpc at rostral and caudal level in wild-type and *Tal1*^*cko*^ brains. **e** Normal number of TH^+^ neurons in in the *Tal1*^*cko*^ mice, both in the substantia nigra pars compacta and the ventral tegmental area (for SNpc, *N* = 3, *p* = 0.84; for VTA, *N* = 3, *p* = 0.77, *t*-test). Scale bar: 100 µm. SNpc: substantia nigra pars compacta; SNpr: substantia nigra pars reticulata; TH: tyrosine hydroxylase; VTA: ventral tegmental area. **f**–**h** Dopamine and metabolite levels in the nucleus accumbens measured in histological samples of the *Tal1*^*cko*^ and wild-type mice, **p < 0.01, *t*-test, *N* = 14 WT, 8, KO). **i**–**j** Dopamine release in the nucleus accumbens subregions. Burst stimulation revealed a dampened dopamine release in the NAc shell of the *Tal1*^*cko*^ mice analyzed by cyclic voltammetric measurement (ANOVA, genotype, for shell F1,26 = 4.06, *p* = 0.041; for core, F1,44 = 0.051, *p* > 0.05). Burst stimulation protocol evoked larger dopamine release than single pulse both in shell and core subregions (ANOVA, stimulus type, for shell, F1,26 = 14.59, *p* < 0.001; for core, F1,44 = 7.09, *p* = 0.011). *p < 0.05, the *Tal1*^*cko*^ mice versus wild-type mice, Bonferroni’s test, *N* = 14 WT, 16 KO, for shell; *N* = 22 WT, 26 KO, for core). **k** Amphetamine-induced non-stimulated dopamine release in the dorsal striatum. No difference in cyclic voltammetry-measured dopamine efflux between the *Tal1*^*cko*^ and wild-type mice (*p* > 0.05, *t*-test). *N* = 17 WT, 14 KO. **l** Stimulated dopamine release under amphetamine perfusion in the dorsal striatum. No differences were detected between the *Tal1*^*cko*^ and wild-type mice (ANOVA F1,266 = 0.07, p > 0.05). N = 13 WT, 11 KO. **m** In vivo dopamine microdialysis in the nucleus accumbens. Amphetamine (3 mg/kg, i.p.) was administered to freely-moving *Tal1*^*cko*^ and wild-type mice followed by microdialysis analysis of extracellular dopamine. No difference was found between the mouse lines (*p* > 0.05, *t*-test). *N* = 5 WT, 6 KO. **n** Acute effect of intraperitoneal amphetamine (timing of injection indicated by a vertical dashed line, 3 mg/kg) after 30 min of habituation in the open field. Both male and female wild-type mice responded with increased locomotion after the injection of amphetamine, whereas amphetamine rescued the hyperactive phenotype in the *Tal1*^*cko*^ mice of both sexes (RM ANOVA genotype F1,68 = 9.6, *p* = 0.0028, time F23,1564 = 11.8, *p* < 0.0001, genotype × time interaction F23,1564 = 3.3, *p* < 0.0001). ***p < 0.001, genotype × amphetamine interaction, ANOVA. *N* = 72 (Amphetamine–6 WT M; 8 KO M; 13 WT F; 12 KO F; Saline–7 WT M; 5 KO M; 12 WT F; 9 KO F). **o** Amphetamine-induced stereotypy was not detected in the wild-type and *Tal1*^*cko*^ mice (ANOVA drug F1,16 = 3.3, *p* = 0.09, genotype F1,16 = 0.8, *p* = 0.37), *N* = 10 WT, 10 KO. **p** Dopamine transporter inhibition by GBR12909 decreased hyperactivity in the *Tal1*^*cko*^ mice (ANOVA dose F2,42 = 15.5, *p* < 0.001; genotype F1,42 = 55.0, *p* < 0.001; interaction F2,42 = 27.2, *p* < 0.001). ***p < 0.001, between the genotypes, Bonferroni’s test; ^###^*p* < 0.001, between vehicle and GBR12909-treated groups, Bonferroni’s test. *N* = 24 WT, 24 KO. **q** Noradrenaline reuptake inhibitor atomoxetine reduced hyperactivity in the *Tal1*^*cko*^ mice (ANOVA dose F2,39 = 6.5, *p* = 0.004; genotype F1,39 = 49.8, *p* < 0.001; interaction F2,39 = 4.4, *p* = 0.02). ***p < 0.001, between the genotypes, Bonferroni’s test; ^###^*p* < 0.001, between vehicle and atomoxetine-treated groups, Bonferroni’s test. *N* = 20 WT, 25 KO. **r** Amphetamine-conditioned place preference. Locomotor activity during the conditioning trials with vehicle (CS-trials) and amphetamine (CS + trials). Amphetamine acutely increased locomotor activity of the wild-type mice, but not that of the *Tal1*^*cko*^ mice (ANOVA genotype × drug interaction F1,60 = 75.66, *p* < 0.001), ^###^*p* < 0.001, between vehicle and amphetamine-treated wild-type mice, *t*-test. Amphetamine caused locomotor sensitization in the wild-type mice but decreased locomotor activity in the *Tal1*^*cko*^ mice to the level of vehicle-treated wild-type mice (ANOVA genotype × drug × conditioning trial interaction F3,180 = 23.36, *p* < 0.001). ^SSS^*p* < 0.001 wild-type mice, between 1st and 4th conditioning trial, Bonferroni’s test; **p < 0.01, between vehicle and amphetamine-treated *Tal1*^*cko*^ mice, *t*-test. *N* = 16 WT, 16 KO. **s** In the conditioned place preference test trial, both the wild-type and the *Tal1*^*cko*^ mice expressed amphetamine-induced conditioned place preference (ANOVA conditioning subgroup F1,28 = 24.27, *p* < 0.001) equally (ANOVA genotype F1,28 = 2.22, *p* = 0.15; ANOVA genotype × conditioning subgroup interaction F1,28 = 0.06, *p* = 0.81). **p < 0.01 between stripe+ and stripe− conditioning subgroups, Bonferroni’s test, *N* = 16 WT, 16 KO. Mice in the stripe+ conditioning subgroup had received amphetamine paired with the striped floor and vehicle paired with the dot floor, while the mice in the stripe− conditioning subgroup had received amphetamine paired with the dot floor and vehicle paired with the striped floor. Data is expressed as time spent in seconds on the striped floor type.

We next asked whether a change in the GABAergic and glutamatergic components of the dopaminergic circuitry, due to the inactivation of *Tal1*, could affect the dopamine neuron numbers. Quantification of dopamine neurons in the ventral midbrain revealed no change in their numbers in the *Tal1*^*cko*^ mice (Fig. 5a–e). We then addressed the functionality of the dopamine system. Analysis of the neurotransmitter levels in the tissue of the dorsal striatum, nucleus accumbens (NAc), and prefrontal cortex, revealed a decrease in the levels of dopamine and its metabolites in the *Tal1*^*cko*^ mice (Fig. 5f–h, Fig S3b–g). All the target tissues of the dopaminergic system showed the same trend, but the difference was most pronounced in the NAc. Serotonin and its metabolite 5-HIAA levels in the dorsal striatum, nucleus accumbens, and prefrontal cortex were unaltered in the *Tal1*^*cko*^ mice (Fig S4). Noradrenaline content was also not changed in the prefrontal cortex of the *Tal1*^*cko*^ mice (Fig S4).

We then used cyclic voltammetry to measure the electrically stimulated dopamine release in brain slices containing the NAc shell, the NAc core, and the dorsal striatum. While the single pulse-evoked dopamine release was normal in the *Tal1*^*cko*^ tissue, burst stimulation protocol revealed a deficit in the release in the *Tal1*^*cko*^ tissue, specifically in NAc shell (Fig. 5i, j, Fig S3h). Clearance of dopamine was unaltered in the *Tal1*^*cko*^ striatal tissue (Fig S3i–k). In amphetamine-induced dopamine efflux in the dorsal striatum, we observed no difference in the magnitude or kinetics of dopamine release between the wild-type or *Tal1*^*cko*^ tissue (Fig. 5k, l). Because of the high variability in the occurrence and levels of amphetamine-induced dopamine efflux in the NAc area, we were not able to perform similar experiments there. Finally, we studied the effect of amphetamine on dopamine release in vivo. Using microdialysis in the NAc and dorsal striatum, we observed unaltered levels of extracellular dopamine and normal amphetamine-induced dopamine release in the *Tal1*^*cko*^ mice (Fig. 5m, Fig S3l).

### Amphetamine ameliorates hyperactivity in the *Tal1*^*cko*^ mice

In order to test the behavioral consequences of pharmacologically induced dopamine signaling in the *Tal1*^*cko*^ mice, we first treated them with amphetamine. Strikingly, in contrast to the wild-type mice, whose locomotor activity was markedly enhanced, the activity of the *Tal1*^*cko*^ mice was decreased by amphetamine (Fig. 5n, r). No amphetamine-induced stereotypy was detected with the dose used (3 mg/kg, Fig. 5o).

Since amphetamine is known to act through multiple targets, we screened the pharmacological mechanisms of the hyperactivity-lowering amphetamine effect in the *Tal1*^*cko*^ mice by using various ligands. Inhibition of the dopamine transporter by GBR12909 increased locomotor activity in the wild-type mice, but decreased hyperactivity of the *Tal1*^*cko*^ mice (Fig. 5p). The noradrenaline transporter inhibitor atomoxetine also decreased hyperactivity in the *Tal1*^*cko*^ mice without an effect on wild-type animals (Fig. 5q). Dopamine D1 and D2 receptor antagonists had no effect on the *Tal1*^*cko*^ mice locomotor activity, but decreased wild-type activity with the highest doses (Fig S3m, n).

### Amphetamine is rewarding in the *Tal1*^*cko*^ mice

To analyze amphetamine-induced reward in the *Tal1*^*cko*^ mice, we carried out an amphetamine conditioned place preference test. We found that amphetamine equally elicited place preference in the wild-type and the *Tal1*^*cko*^ mice (Fig. 5r, s). When we analyzed the effect of amphetamine on locomotor activity during the conditioning, we observed that whereas amphetamine caused a typical locomotor sensitization in the wild-type mice, it decreased the locomotor activity in the *Tal1*^*cko*^ mice to the level of vehicle treated wild-type mice (Fig. 5r).

## Discussion

GABAergic and glutamatergic neurons in the anterior brainstem have been implicated in the regulation of mood, motivation and movement. These neurons are developmentally and anatomically highly complex, and some of their subtypes control the brainstem neuromodulatory systems. We show that the *Tal1*^*cko*^ mice, with changes in specific neuronal subgroups in the anterior brainstem, have alterations in their activity, impulsivity and reward behavior, that recapitulate many endophenotypes of ADHD. Although some of the *Tal1* dependent neurons are thought to control the dopaminergic system, the levels of dopamine and its release were only modestly affected in the *Tal1*^*cko*^ mice. Strikingly, the behavioral response of the *Tal1*^*cko*^ mice to amphetamine resembled that of ADHD patients. Our studies suggest *Tal1* dependent anterior brainstem GABAergic and glutamatergic neuron subgroups as an important new focus for understanding the neurobiology of this common behavioral disorder.

### Behavioral defects in the *Tal1*^*cko*^ mice: comparison with ADHD

An earlier study of mice with a global neuronal inactivation of the *Tal1* gene (*Nestin-Cre*; *Tal1*^*flox*^) suggested a hyperactivity phenotype, but the analysis was complicated by defects in locomotion, circling behavior and premature lethality^11^. Our approach to use the *Tal1*^*cko*^ mice allowed a detailed behavioral characterization of mice, in which the neuronal defects due to *Tal1* deficiency are restricted to specific regions in the anterior brainstem. The behavioral changes in the *Tal1*^*cko*^ mice include hyperactivity, enhanced motor impulsivity, altered reward processing, deficits in learning, behavioral flexibility and task completion—all cardinal features of ADHD.

The *Tal1*^*cko*^ mice display a specific hyperactive phenotype comprising initial normoactivity in a novel environment that rapidly develops into hyperactivity^23^. The hyperactive phenotype is present already in a juvenile age, and persists into adulthood. Remarkably, the *Tal1*^*cko*^ mice exhibit normal spontaneous locomotor activity during the light period of their circadian cycle, consistent with normal night-time activity by actigraphy observation in patients with ADHD^24^. This suggests that the observed hyperactivity in *Tal1*^*cko*^ mice is not generalized, but instead shows specificity in tasks that require exploration and attention. Interestingly, different forms of impaired habituation have been reported in ADHD patients^25,26^ in addition to some other psychiatric disorders such as schizophrenia^27^. The prepulse inhibition of the startle response, used to measure defective sensorimotor gating in schizophrenia^28^, appears normal in ADHD patients^28,29^ (but see^29^ and^30^), and also was unchanged in the *Tal1*^*cko*^ mice. In the analyses of habituation of the *Tal1*^*cko*^ mice, we were able to distinguish deficiencies both in intra-session and inter-session habituation, which may provide means for refinement of the habituation deficit as an endophenotype in ADHD^31,32^. As with hyperactivity, initially normal impulsive behavior in ADHD patients manifests as motor impulsiveness as the test session progresses^23^. This pattern is also phenocopied in the *Tal1*^*cko*^ mice. The *Tal1*^*cko*^ mice are hypersensitive to rewarding effect of saccharin, which is of interest as ADHD and substance use disorder share comorbidity^33^. Normal social interaction was observed in the *Tal1*^*cko*^ mice, unlike ADHD patients that have been reported to show changes in their social interaction^34^.

### Putative relationships between the neuroanatomical and behavioral changes in the *Tal1*^*cko*^ mice

*Tal1* dependent GABAergic neurons in the VTA, RMTg and SNpr regulate the adjacent dopaminergic neurons, but also have projections to other brainstem nuclei and more anterior regions of the brain, participating in the control of both motivated behavior and movement. For example, the targets of the VTA GABAergic neurons include the PFC, implicated in the control of impulsive behavior^6^, and the nucleus accumbens, which has been implicated in reward behavior^4^. PFC also receives neuromodulatory afferents from the brainstem nuclei which are innervated by the *Tal1* dependent GABAergic neurons^35^. Of these, locus coeruleus noradrenergic and dorsal raphe serotonergic systems have been linked to impulsivity^8^. The RMTg controls avoidance behavior^3,36^, but a lesion or inhibition of the RMTg also results in increased locomotor activity in the rat^37–40^. The SNpr GABAergic neurons provide output from the basal ganglia inhibiting the motor nuclei in the thalamus. Therefore, loss of SNpr neurons may contribute to the hyperactivity of the *Tal1*^*cko*^ mice. The neurons of the SNpr fall into two broad categories, the anterior SNpr and the posterior SNpr neurons, which differ by their embryonic origins and gene expression^10^. In the *Tal1*^*cko*^ mice, only the posterior-type SNpr neurons are affected. Future studies should address the projection patterns and functions of the distinct SNpr subgroups, including projections to the thalamic nuclei. In addition to GABAergic neurons associated with the dopaminergic nuclei, *Tal1* dependent GABAergic neurons are located in the ventral tegmental nucleus of Gudden (VTg)^11^^,12^. These neurons project to the medial mammillary bodies, regulate hippocampal theta waves, and are important for memory^13^. Interestingly, lesions of the VTg in rodents also cause hyperactivity^41,42^.

Whereas brainstem GABAergic nuclei are defective in the *Tal1*^*cko*^ mice, glutamatergic neurons in specific brainstem nuclei, including the interpeduncular nucleus, LDTg and SNpc, are increased in their numbers^10^. In contrast to the inhibitory RMTg projections, the LDTg sends excitatory glutamatergic projection to the VTA to regulate reward behavior^15^. The increased numbers of the glutamatergic neurons may enhance excitatory drive to their target structures. However, although changes in excitation-inhibition balance have been associated with hyperactivity and ADHD^43–46^, it remains unclear how the imbalance in the brainstem GABAergic and glutamatergic neuron differentiation is reflected in the synaptic control of the multiple target cell types of the GABAergic and glutamatergic brainstem neurons in the *Tal1*^*cko*^ mice.

It is unclear if the development or function of these tegmental nuclei is altered in the human ADHD patients. Very interestingly, however, a reduction in the anterior brainstem size has been shown to distinguish the ADHD patients from the controls^47,48^. Further studies should address if this reduction is due to changes in the local tegmental nuclei, axonal tracts, or both.

### Dopamine signaling in the *Tal1*^*cko*^ mice

Many of the *Tal1* dependent anterior brainstem neurons are thought to regulate the dopaminergic neurons and basal ganglia circuits, whereas altered basal ganglia activity and dopaminergic signaling has been associated with ADHD. However, the exact roles of these pathways have remained elusive and it has been debated whether ADHD is a hypodopaminergic or hyperdopaminergic defect^2^.

Based on the neuroanatomical defects in the *Tal1*^*cko*^ mice, an increase in the dopamine neuron activity could be predicted. However, our neurochemical analyses of the *Tal1*^*cko*^ mice suggest only minor changes in the dopaminergic neurotransmission. Moreover, in the target tissues, the dopamine and dopamine metabolite levels are decreased rather than increased. Our results are consistent with the conclusion that ADHD-like symptoms can correlate with apparently reduced dopaminergic signaling. It is possible that the dampening of dopamine signaling in the *Tal1*^*cko*^ mice is due to developmental feedback regulation or functional compensation.

Stimulant drugs such as amphetamine provide the mainstay of the ADHD pharmacotherapy^49^. Hyperactivity in the *Tal1*^*cko*^ mice was rescued by amphetamine treatment, a finding that further supports the putative ADHD-like phenotype. Amphetamine did not induce stereotypy by the moderately low dose used, which is in line with earlier reports^50,51^. Striatal dopamine system may be underactive in the ADHD patients, and stimulant medication restores the deficit^52^. Similarly, amphetamine increases extracellular dopamine in the striatum, and at the behavioral level normalizes the hyperactive state in the *Tal1*^*cko*^ mice. The release mechanisms for dopamine also remain functional in the *Tal1*^*cko*^ mice both in vitro and in vivo, as shown by voltammetry and microdialysis data, respectively.

The mechanism of action of amphetamine in relieving ADHD symptoms remains elusive, partly because the pathophysiological basis of ADHD has not been confirmed, and partly because amphetamine’s effects are mediated by multiple molecular targets. Our data using more selective pharmacology suggests that, at least in the *Tal1*^*cko*^ mice, amphetamine may act through increasing both dopamine and noradrenaline tone. Dopaminergic tone can be increased directly by stimulants both in prefrontal and striatal regions^52^. However, noradrenaline very sparsely innervates striatum, but instead the brainstem noradrenergic neurons send projections to the prefrontal regions^53^, and consequently prefrontal cortex has been suggested as a target for the atomoxetine effect^54^. Dopamine receptor antagonists failed to rescue the hyperactivity of the *Tal1*^*cko*^ mice, which supports a conclusion that *Tal1*^*cko*^ mice have a hypofunctional dopamine system. Taken together, stimulant-sensitive hypodopaminergia of the *Tal1*^*cko*^ mice points towards a view of co-existence of an ADHD-like phenotype with attenuated dopamine signaling.

Our observation that amphetamine can have dramatically different effect on the locomotor activity and its sensitization in the wild-type and *Tal1*^*cko*^ mice, yet is reward-inducing for both of them, further suggests that the hyperactivity and reward are independent of each other and mediated by distinct circuits. Interestingly, it has been proposed that tonic and phasic dopamine signaling are differentially affected in the ADHD patients^55^.

### Comparison with other animal models of ADHD

ADHD is likely a heterogeneous deficit with different neurobiological bases. Similarly, clinical symptoms of ADHD have been observed in a variety of gene-modified animal strains, which have been adopted as animal models of ADHD^2^. Dopamine transporter deficient mouse line, coloboma mutant mice, and spontaneously hypertensive rats show behavioral hyperactivity similar to the *Tal1*^*cko*^ mice, but there are also specific differences between these models^56^. In *Tal1*^*cko*^ mice the task-related hyperactivity develops over time, which is similar in spontaneously hypertensive rats, but this is not seen in dopamine transporter deficient mouse line which are highly hyperactive from the beginning of the task. Coloboma mutant mice harbor a selection of neurological and other symptoms, which downplay their utilization as an animal model of ADHD^56^. The hyperactivity of *Tal1*^*cko*^ mice shows sensitivity to stimulants, which is similar to dopamine transporter deficient mouse model and coloboma mouse model^57,58^, but spontaneously hypertensive rats have shown variable responses^59–62^. Atomoxetine reduces hyperactivity in *Tal1*^*cko*^ mice and in spontaneously hypertensive rats, but not in dopamine transporter deficient mouse line^63,64^. Differences between the models exist in prepulse inhibition, which is deficient in spontaneously hypertensive rats^65^ and in the dopamine transporter deficient mouse line^66^, while *Tal1*^*cko*^ mice are normal in this aspect. Social interaction was found to be normal in *Tal1*^*cko*^ mice, while dopamine transporter deficient mice show decreased social investigation and increased reactivity in response to social investigation^67^. In spontaneously hypertensive rats, an unchanged^68^, reduced^61,68^, or increased^69^ social interaction has been reported. Impulsiveness is a shared phenotypic property in *Tal1*^*cko*^ mice and spontaneously hypertensive rats. Both of these animal models have hypodopaminergic neurochemical phenotype^70^. It is not clear how well any of these animal models satisfy the construct validity of human ADHD. Multiple preclinical models may be needed to fully understand this behavioral disorder.

## Conclusion

ADHD is a highly heritable neurodevelopmental disorder, but its genetic components remain poorly understood. Although work with genetically modified mice has demonstrated the causal relationship between the altered dopaminergic neurotransmission and hyperactivity, the candidate gene studies have failed to reveal a substantial role for variation in genes involved in dopaminergic neurotransmission as the genetic basis for ADHD^2^. Therefore, it is possible that defects in the other components of the basal ganglia circuitry are behind the etiology of this disorder. Although *Tal1* gene variants have not been associated with the etiology of ADHD, our results show that a defect in a specific developmental event dependent on a single transcription factor, namely balanced differentiation of GABAergic and glutamatergic neurons in the developing anterior brainstem, leads to several endophenotypes of ADHD. Studies of these neurons, and the circuits they participate in, can lead to better understanding of the normal and abnormal control of activity, attention and reward.

## Supplementary information

Supplemental material
