# Supplementary material for: ADHD-like behaviors caused by inactivation of a transcription factor controlling the balance of inhibitory and excitatory neuron development in the mouse anterior brainstem
Source: Transl Psychiatry. 2020 Oct 21;10:357. doi: 10.1038/s41398-020-01033-8 (PMC7578792; doi:10.1038/s41398-020-01033-8)
Supplement: Supplementary file 1 — Supplemental material [file 41398_2020_1033_MOESM1_ESM.docx]

SUPPLEMENTARY INFORMATION

Supplementary Table

Table 1

Supplementary Figures

**Supplementary** **Fig S1.**

A. Rotarod test. The *Tal1^cko^* mice had longer latency to fall (F1,28=10.7, p=0.0028). B. Multiple rods. The *Tal1^cko^* mice had longer latency to turn (F1,28=20.9, p<0.0001) and end the trial (F1,28=23.1, p<0.0001). **p<0.01, ***p<0.001, between genotypes, ANOVA.

**Supplementary Fig S2.**

A. Novel object recognition. Preference for the novel object was equal between the *Tal1^cko^* and wild-type mice (F1,32=0.001, p=0.97).
B. Novel object recognition. Distance moved was higher in the *Tal1^cko^* than in the wild-types both in training (F1,30=7.7, p=0.0093), and during the test trial (F1,32=20.6, p<0.0001).
C. Fear conditioning. Contextual learning was impaired in the *Tal1^cko^* mice (freezing during the context trial, ANOVA genotype F1,49=10.4, p=0.0022; ANOVA sex F1,49=7.6, p=0.008; ANOVA genotype x sex interaction F1,49=0.22, p=0.64). **p<0.01, between genotypes, *t*-test.

**Supplementary Fig S3.**

A. Locomotor activity during the CPP test trial, in which the *Tal1^cko^* mice moved more than the wild-type mice, *p<0.05, *t*-test.

B-D. Equal tissue dopamine and metabolite levels in the dorsal striatum between the wild-type and *Tal1^cko^* mice (p>0.05, *t*-test).

E-G. Equal tissue dopamine and metabolite levels mice in the prefrontal cortex between the wild-type and *Tal1^cko^* mice (p>0.05, *t*-test).

H. Dopamine release evoked by single pulse or burst stimulation in the dorsal striatum by cyclic voltammetry was not changed in the *Tal1^cko^* mice (p>0.05, ANOVA).

I-K. Dopamine clearance half-life (T1/2) in cyclic voltammetry was found to be normal in the *Tal1^cko^* mice after single pulse and burst protocols (p>0.05, ANOVA).

L. Amphetamine (3mg/kg i.p.) induced equal dopamine release in the dorsal striatum in the wild-type and Tal1cko mice as measured by in vivo microdialysis, p=0.92, t-test.
M. Dopamine D1 receptor antagonism by SCH23390 failed to modulate locomotor activity in the *Tal1^cko^* mice (ANOVA dose F2,39=0.4, p=0.7; genotype F1,39=46.4, p<0.001). In wildtype mice, SCH23990 decreased locomotor activity (ANOVA dose F2,16=7.9, p=0.004; **p<0.01, between genotypes; ^##^p<0.01, between vehicle and SCH 0.1, Bonferroni’s test).

N. Dopamine D2 receptor antagonism by raclopride did not change locomotor activity of the *Tal1^cko^* mice (ANOVA dose F3,35=0.6, p=0.62; genotype F1,35=112.7, p<0.001). In wildtype mice, raclopride decreased locomotor activity (ANOVA dose F3,18=13.4, p<0.001; ***p<0.01, between genotypes; ^###^p<0.001, between vehicle and raclopride 20, Bonferroni’s test).

**Supplementary Fig S4.**

A-B. Equal tissue 5-HT and 5-HIAA levels in the dorsal striatum between wild-type and *Tal1^cko^* mice (p>0.05, *t*-test).

C-D. Equal tissue 5-HT and 5-HIAA levels in the nucleus accumbens between wild-type and *Tal1^cko^* mice (p>0.05, *t*-test).

E-F. Equal tissue 5-HT and 5-HIAA levels in the prefrontal cortex between wild-type and *Tal1^cko^* mice (p>0.05, *t*-test).

G. Equal tissue noradrenaline levels in the prefrontal cortex between wild-type and *Tal1^cko^* mice (p>0.05, *t*-test).

Supplementary Materials and methods

**Animals**

Animal care and housing: Animals were maintained in the animal facility of the University of Helsinki, under standard laboratory conditions (21±2 °C, 12-hour light-dark cycle, lights on at 6 AM) with free access to food and water. The mice were housed in the individually ventilated cages (groups of 2-5 mice/cage; Mouse IVC Green Line – overall dimensions 391 x 199 x 160 mm, floor area 501 cm^2^; Tecniplast, Italy) with half of the cage covered by wire bar food hopper. Air inlet and outlet valves were located in the cage lid, on top of the cage. The bedding (aspen chips 5 × 5 × 1 mm, Tapvei Oy, Finland) was changed weekly. Nesting material (aspen strips, PM90L/R, 3 mm × 20 cm, Tapvei Oy, Finland) and wooden block (100 x 20 x 20 mm, Tapvei Oy, Finland) were provided as environmental enrichment.

**Behavioral analyses**

Open field (OF).

The mice were released in the corner of novel open field arena (white floor, transparent walls, 30 x 30 cm, Med Associates). Horizontal and vertical activity (distance moved and rearings, respectively) was recorded by infrared sensors during 30 minutes (light intensity ~150 lx). Peripheral zone was defined as a 6 cm wide corridor along the wall. For amphetamine-induced locomotion, the mice were habituated to the arena for 30 minutes, then injected intraperitoneally with 3 mg/kg amphetamine and monitored for 90 minutes. For testing of habituation, the female mice of the last cohort were tested repeatedly in OF on 5 subsequent days (OF5d), with interval of 24 hours.

Elevated zero maze (EZM).

The maze was made of grey plastic and had an annular runway (diameter 50 cm, width 5 cm). Two opposing 90° sectors of the runway were protected by an inner and outer wall of grey polyvinyl‐ chloride (height 15 cm). The two remaining sectors were unprotected (open zones). The apparatus was mounted 40cm above the floor and exposed to indirect and diffuse room light (~25 lux). The mouse was placed in the middle of the one of the closed sectors and allowed to explore the maze freely for 5 min. Distance travelled and time spent in different areas (open, closed) was recorded with Ethovision XT 10 tracking equipment (Noldus, Netherlands).

Light-dark box (LD).

The test was carried out in the open field arena (30 x 30 cm, Med Associates, St. Albans, VT) equipped with infrared light sensors detecting horizontal and vertical activity. The dark insert (non-transparent for visible light) was used to divide the arena into two halves, an opening (a door with a width of 5.5 cm and height of 7 cm) in the wall of the insert allowed animal’s free movement from one compartment to another. Illumination in the center of the light compartment was ~550 lx (bright ceiling lights). Animal was placed in the dark compartment and allowed to explore the arena for 10 minutes. Distance travelled, number of rearings, latency to enter the light compartment and time spent in different compartments were recorded by the program. The number of faecal boli was counted by experimenter after the end of trial.

Social approach (SOC).

A square box (40 x 40 cm, side walls 50 cm high) was used as an arena, where in the middle of one side wall a perforated Plexigals cylinder was placed. The test mouse was allowed to explore the arena for 5 minutes (habituation) and removed then. An unfamiliar CD-1 mouse was placed then in the cylinder and test mouse returned to the arena for additional 5 minutes (social approach). The distance travelled and time spent in exploring the cylinder was measured during both phases (Ethovision XT 10).

3-compartment test for sociability (3COMP).

The test apparatus consisted of three rectangular compartments (18 x 35 x 18 cm) divided by Plexiglas walls with openings (6 x 5 cm) allowing the animal to move between the compartments. Both side compartments contained an empty transparent Plexiglas holder (8 cm diameter, 10 cm high). The test mouse was first released in the central compartment and was allowed to habituate to the apparatus for 10 min. An unfamiliar sex- and age-matched CD-1 mouse (stranger) was placed in one of the holders (with small holes allowing a snout contact between the animals but not biting or other fighting behavior). The location of the stranger mouse in either of the two holders varied systematically between the trials. The test mouse was then allowed to explore the whole apparatus for 10 min. The time spent in and entries into each compartment were recorded. In addition, the time spent in the zone around the holders (4 cm wide) was measured (Ethovision XT 10).

Rotarod (RR).

The accelerating rotarod (Ugo Basile, Comerio, Italy) test was performed on two consecutive days. The mice were given three trials a day with an inter-trial interval of 1 hour. Acceleration speed from 4 to 40 rpm over a 5-min period was chosen. The latency to fall off was recorded with the cut-off time set at 6 min.

Multiple static rods (MSR).

A series of five 60 cm long wooden rods of decreasing diameters (rod 1 - 27 mm; rod 2 - 21 mm; rod 3 - 15 mm; rod 4 - 11 mm; and rod 5 - 8 mm) was used, each perpendicularly fixed at one end to the table. The apparatus was elevated 60 cm above a soft surface. The mouse was placed at 2 cm from the distal end of the rod, facing towards open end of the rod. The latencies to turn 180° to face the fixed end of the rod, and then to travel 60 cm to the table, were recorded. Cut-off time was set at 2 minutes.

Hot-plate (HP).

Standard hot plate (Ugo Basile) was heated to 52°C and the mouse was confined there by Plexiglas cylinder (diameter 19 cm, height 26 cm). The latency to display licking or shaking of the hindpaw was recorded.

Pre-pulse inhibition (PPI).

Mice were enclosed in a transparent plastic tube (Ø 4.5 cm, length 8 cm) that was placed in the startle chamber (Med Associates) with a background white noise of 65 dB and left undisturbed for 5 minutes. Testing was performed in 12 blocks of 5 trials and five trial types were applied. One trial type was a 40-ms, 120-dB white noise acoustic startle stimulus (SS) presented alone. In the remaining four trial types the startle stimulus was preceded by the acoustic prepulse stimulus (PPS). The 20-ms PPS were white noise bursts of 68, 72, 76 and 80 dB. The delay between onset of PPS and SS was 100 ms. The 1st and 12th block consisted of SS-alone trials. In remaining blocks the SS and PPS+SS trials were presented in pseudorandomized order such that each trial type was presented once within a block of 5 trials. The inter-trial interval ranged between 10 and 20 seconds. The startle response was recorded for 65 ms starting with the onset of the startle stimulus. The maximum startle amplitude recorded during the 65-ms sampling window was used as the dependent variable. The startle response was averaged over 10 trials from blocks 2-11 for each trial type. The prepulse inhibition for each PPS was calculated by using the following formula: 100-[(startle response on PPS+SS trials / startle response on SS trials) x 100].

Forced swim test (FST).

The mouse was placed for 6 minutes in the glass cylinder (Ø 18 cm, height 25 cm) filled with water at 23 ± 1 °C to the height of 15 cm. The time of immobility (passive floating, when the animal was motionless or doing only slight movements with tail or one hind limb, whereas the animal was judged to be active when struggling, climbing or swimming using all four paws) was measured in 2 min intervals (Ethovision XT 10).

Fear conditioning (FC).

The experiments were carried out employing a computer-controlled fear conditioning system (TSE Systems, Germany). Training was performed in a transparent acrylic cage (23 × 23 × 35 cm) within a constantly illuminated (~ 100 lx) fear conditioning box. A loudspeaker provided a constant, white background noise (68 dB) for 120 s followed by 10 kHz tone (CS, 76 dB, pulsed 5 Hz) for 30 s. The tone was terminated by a footshock (US, 0.6 mA, 2 s, constant current) delivered through a stainless steel grid-floor (bar Ø 4 mm, distance between the bars 10 mm). Two CS-US pairings were separated by a 30 s pause. Contextual memory was tested 24 h after the training. The animals were returned to the conditioning box and total time of freezing (defined as an absence of any movements for more than 3 s) was measured through infrared light beams scanned continuously with a frequency of 10 Hz. The CS was not used during this time. Memory for the CS (tone) was tested 2 h later in a novel context. The new context was a similarly sized acrylic box with black non-transparent walls and smooth floor. A layer of wood chips (clean bedding material) under the floor provided a novel odour to the chamber. After 120 s of free exploration in a novel context the CS was applied for additional 120 s and freezing was measured as above.

T-maze (TM).

The T-maze was made of grey PVC. Each arm measured 30 cm×10 cm. A removable central partition extended from the center of the goal wall of the T to 7 cm into the start arm preventing the mouse from seeing or smelling the non-chosen arm during the sample run, thus minimizing interfering stimuli. The entrance to each goal arm was fitted with a guillotine door. Each trial consisted of an information-gathering, sample run, followed immediately by a choice run. For the sample run a mouse was placed in the start arm, facing away from the choice point with the central partition in place. It was allowed to choose a goal arm and confined there for 30 s by lowering the guillotine door. Then the central partition was removed, the mouse replaced in the start arm, and the guillotine door was raised. Alternation was defined as entering the opposite arm to that entered on the sample trial (whole body, including tail). Three trials were run per day with an inter-trial interval at least 30 min, on two consecutive days (6 trials altogether).

Water maze (WM).

The equipment consisted of a black circular swimming pool (Ø 120 cm) and an escape platform (Ø 10 cm) submerged 0.5 cm under the water surface in the centre of one of four imaginary quadrants. The animals were released to swim in random positions facing the wall and the time to reach the escape platform (maximum time 60 s) and the swimming distance were measured in every trial (Ethovision XT 10). In addition, thigmotaxis, the time spent swimming within the outermost ring of the pool (10 cm from the wall) was measured. Two training blocks consisting of three trials each were conducted daily. The interval between trials was 4-5 min and between training blocks about 5 hours. The hidden platform remained in a constant location for 3 days (6 initial training sessions) and was thereafter moved to the opposite quadrant for 2 days (4 reverse training sessions). The probe trials were conducted approximately 18 h after the last initial and reverse training sessions. The mice were allowed to swim in the maze for 60 seconds without the platform available. Spatial memory in the probe trials was estimated by preference of swimming in the trained region (imaginary circular area of Ø 30 cm, around the previous platform location) over swimming in corresponding regions in the three other quadrants. After the 2nd probe trial, the mice were tested for one block of 3 trials with the platform made visible in the quadrant not employed previously.

Circadian activity (CIRC).

The InfraMot system (TSE, Germany) was used for registering total activity of a single-housed animals and measured by the displacement of body-heat image (infrared radiation) over time. The mice were housed in Type II cages (267 mm x 207 mm x 140 mm) with bedding and nesting material (aspen chips, Tapvei and Nestlet, Ancare), with the sensor assembly mounted on the cage cover. The recording continued for 7 days. Water was provided in 15 ml Falcon tubes (with cut tip), two tubes were available side-by-side on each cage cover. Tubes were weighed and refilled every day, weight difference was considered as liquid consumption. For saccharin preference test (SAC), one of the tubes was filled with 0.5%-saccharin solution, and saccharin preference was calculated as percentage of consumed saccharin from total (saccharin + water) consumption.

Nest construction (NEST).

The mice were placed in individual testing cages (Type II, 267 x 207 x 140 mm) containing regular bedding and one hour before the dark phase a Nestlet of 2.5 g compressed cotton (Ancare, Bellmore, NY) was added into the cage. After 12 h the nests were assessed on a rating scale of 1-5: 1 = Nestlet >90% intact, 2 = Nestlet 50-90% intact, 3 = Nestlet mostly shredded but no identifiable nest site, 4 = identifiable but flat nest, 5 = crater-shaped nest.

Burrowing (BURR).

Three hours before the beginning of dark period the mice were placed in individual test cages (Type III, 425 x 266 x 155 mm) containing regular bedding and burrowing tube (a grey plastic tube, 6.3 cm inner diameter, closed from one end, open end was supported by 2 metal bolts, thus raised at 3 cm above the floor) filled with ~250 g of regular food pellets. The amount of food digged out of the tube was measured after 3 and 14 hours.

Marble burying test (MBT).

The cage preparation involved filling clean cages (30×15×12 cm) with 4-5-cm chipped aspen bedding (Tapvei). The mice were allowed to habituate to the cage for 10 min and then removed briefly. Twenty glass marbles (15 mm diameter) were gently placed on bedding equidistantly in a 4×5 arrangement. The mice were returned to the cage and the number of marbles buried (>50% of the marble covered by bedding material) during 30 minutes was recorded.

Grooming (GRO).

Each subject was placed individually in a clean standard mouse cage (without bedding) and allowed to adapt for 10 min. Following this habituation period, subjects were observed for another 10 min, and cumulative time spent in self-grooming of all body regions was scored and recorded by experimenter.

Stress-induced hyperthermia (SIH).

Stress-induced hyperthermia was assessed by measuring the rectal temperature of the mice twice (rodent thermometer with rectal probe for mice, Bioseb, France). The first measurement served as recording of baseline and as a stressor at the same time. The second measurement was done 10 min later to reveal the stress-induced hyperthermia reaction.

IntelliCage (IC).

Mice were subcutaneously injected with RFID transponders (Planet ID GmbH, Germany) for individual identification. The IntelliCage by NewBehavior (TSE Systems, Germany) is an apparatus designed to fit inside a large cage (610 x 435 x 215 mm, Tecniplast 2000P). The apparatus itself provides four recording chambers that fit into the corners of the housing cage. Access into the chambers is provided via a tubular antenna (50 mm outer and 30 mm inner diameter) reading the transponder codes. The chamber contains two openings of 13 mm diameter (one on the left, one on the right) which give access to drinking bottles. These openings are crossed by photo beams recording nose-pokes of the mice and the holes can be closed by motorized doors. Four triangular red shelters (Tecniplast, Buguggiate, Italy) were placed in the middle of the IntelliCage and used as sleeping quarters and as a stand to reach the food. The floor was covered with a thick (2-3 cm) layer of bedding. The IntelliCage was controlled by a computer with dedicated software, executing preprogrammed experimental schedules and registering the number and duration of visits to the corner chambers, nose-pokes to the door openings and lickings as behavioral measures for each mouse. In the beginning of the test, the mice were released in the IntelliCage with all doors opened allowing unlimited access to the bottles (free adaptation).

Free adaptation: the mice were allowed to explore new environment with all doors open providing free access to drinking tubes. Exploratory activity (corner visits for the first 1 h and before darkness), locomotor activity (corner visits) as well as circadian activity, anxiety parameters (latency to the first corner visit, nose-poke and licking), drinking behaviour (number of lickings, % of visits with licking) and spontaneous alternations were analysed.

Adaptation to nose-poke: All doors were closed at the beginning of experiment and mice were required to poke into closed gates to reach drinking tubes. Only the first nosepoke of the visit opened the door for 5 seconds (pre-defined time). Animals had to start a new visit in order to get access to water again.

Adaptation to drinking sessions: doors were programmed to open after the first nose-poke only during two 2-hour periods, from 20:00 to 22:00 and from 04:00 to 06:00. Drinking sessions were applied for increasing the motivation to visit the corners and thereby providing defined time windows for testing of learning.

Flexible sequencing task. The animals were assigned two correct corners, which were rewarded alternately during drinking sessions (task acquisition – after visiting a correct corner, the next reward could be obtained in diagonally opposite corner, correct sequence of visits 1-3-1-3 etc., corners 2 and 4 were assigned as incorrect and never rewarded). After 4 days (8 sessions) the sequence was reversed, i.e. reward (water) was delivered in previously incorrect corners for next 8 sessions. After first reversal session, two more reversals were performed. The experiment was performed according to earlier report ^1^.

Motor impulsivity. The motor impulsivity task was used to evaluate the ability of the mice to wait for a certain period of time to access water. In this task, all four corners operated in the same way, 24 h per day. The first nose-poke in a visit determined the correct side and initiated a delay period, followed by 5 s during which 3 green LEDs above the door on the correct side switched on and the door opened for drinking. Any nose-poke during the delay period was considered a premature response, whereas the first nose-poke at the open door was counted as a correct response. Correct response latency was defined as the time that elapsed between the onset of the light stimulus and the correct response. The task had three phases. During the first 2 days, delays were set at 0 s (baseline). Then, the delays varied randomly between 0.5, 1.5, 2.5 and 3.5 s for the rest of the task. During the next 2 days, premature responses had no consequence (training). During the final phase of 7 days (testing), premature responses stopped the trial, requiring the mouse to leave the corner and start a visit again.

Saccharin preference and Delay discounting. In this task, all four corners operated in the same way, 24 h per day: with a given delay after onset of a visit, doors opened spontaneously for a 7-s drinking period. To force a choice of either the left or right bottle, a nose-poke at any open door closed or prevented opening of the door on the other side. The task was divided into three phases. First, with delays set at 0 s, in each corner (two left corners, two right corners), one bottle of water was replaced with 0.5% saccharin and animals were allowed to develop preferences for saccharin bottles over 3 days (training). In the second phase (discounting), delays for opening the saccharin doors were increased by 1 s every 24 h. After 8 days, this resulted in a delay of 8 s. For the final 3 days (extinction), delays were reset to 0 s. A saccharin preference score was calculated as percentage of lick number at saccharin bottles form total lick number (saccharin + water).

Schedule. Three cohorts of the animals were tested in the following schedule, with at least 24 h interval between the tests:

Male mice, Cohort-1 (12 ko, 18 wt): EZM – LD – OF – SOC – RR – HP – MSR – PPI – FST – FC – CIRC – NEST – SAC – AMPH

Male mice, Cohort-2 (7 ko, 9 wt): LD – OF – 3COMP – TM – NOR

Male mice, Cohort-3 (10 ko, 14 wt): OF – WM – CIRC – NEST – SAC – SIH – BURR – MBT

Female mice, Cohort-1 (11 ko, 13 wt): IC – LD – GRO – MBT – BURR – NEST

Female mice, Cohort-2 (7 ko, 13 wt): IC – LD – OF – 3COMP – TM – NOR – CIRC – NEST – OF-HAB – AMPH

Female mice, Cohort-3 (10 ko, 13 wt): IC – OF5d – FC

Amphetamine-induced stereotypy

Mice were injected with amphetamine (3 mg/kg, i.p.), and 30 minutes after the injection they were observed for stereotypic behavior for 2 minutes. The monitored behaviors included head bobbing (up-and-down movements of the head), continuous sniffing, circling, and continuous nail and/or wood chip biting or licking, as described earlier ^2^.

Amphetamine conditioned place preference

Plastic material patterned with stripes or with dots was used on the cage floors as tactile conditioning stimuli (CS). One of the materials was patterned with stripes and the other was patterned with dots. Experiment consisted of three phases: habituation (one session), conditioning (eight sessions), and place preference testing (one session). During all trials activity and location of mice was recorded with imaging system (Ethovision XT 10, Noldus, Netherlands). The light intensity in the conditioning apparatus was set at the level of 44 lux. In the habituation session the mice were given a saline injection and placed into the conditioning apparatus with no floor materials installed for 30 min. In the conditioning sessions mice were randomly assigned to one of the conditioning subgroups (Stripe+ and Stripe-). Mice in the Stripe+ group received amphetamine (3 mg/kg, i.p.) as an unconditioning stimulus (US) paired with stripe patterned floor and saline paired with the dot patterned floor. Mice in the Stripe− group received amphetamine paired with dot patterned floor and saline paired with the stripe patterned floor. After receiving an amphetamine injection (3 mg/kg, i.p., dissolved in saline) the mice were placed in the conditioning apparatus for 30 min. Animals received 4 conditioning trials of each type on alternating days. The order of amphetamine and saline injection was counterbalanced across the groups. Each conditioning cage and floor materials were used once a day for one mouse and cleaned after the daily trial. Place preference test was performed 24 h after the last conditioning session. The conditioning apparatus consisted both CS thus bisecting the floor area into two zones (stripe and dot). Mice were injected with saline and placed at the intersection of the two CS floor materials for 30 min. The left-right position of the floors was counterbalanced within groups. Time spent on the stripe floor was used as a primary dependent variable in the data analysis.

Immunohistochemistry and DA neurons counts.

Adult wild-type (n=3) and *Tal1^En1cko^* (n=3) male mice (3 months old) were anesthetized and transcardially perfused with saline and 4% paraformaldehyde in PBS. Brains were then processed as previously described ^3^, and sectioned at 10 µm with a microtome (Leica). For immunofluorescence, sections were treated with mouse anti-tyrosine hydroxylase (Millipore, #MAB318, 1:600) overnight at 4°C and the day after the immunostaining was visualized with AlexaFluor 488- conjugated secondary antibody raised in donkey (Thermo Scientific) for 3 hours at room temperature. Sections were then counterstained with DAPI and the right side of the section was imaged with an Olympus BX63 microscope connected to a DP72 camera, and processed with Adobe Photoshop CS6. The total number of TH positive neurons was determined using Image J. Counting was performed at 10X, at regular intervals (10-12 sections in total). The data were analyzed with Prism6 (GraphPad) and unpaired, two tailed t-test was used to compare the means across the two groups (significance p<0.05). Results are presented as mean of the TH positive cells per section ± s.e.m.

Measurement of dopamine release by cyclic voltammetry

Male mice at 11-13 weeks of age, were decapitated and 300 µm thick coronal brain slices that contained cortex and striatum were cut on a vibratome (7000 smz-2, Campden Instruments, Leics, England) in ice-cold cutting saline containing 125 mM NaCl, 2.5 mM KCl, 26 mM NaHCO3, 0.3 mM KH2PO4, 3.3 mM MgSO4, 0.8 mM NaH2PO4, and 10 mM glucose. Slices were allowed to recover in holding chamber for 1–2 hours at 32 °C, in oxygen-bubbled (95 % O2, 5 % CO2) recording saline containing 125 mM NaCl, 2.5 mM KCl, 26 mM NaHCO3, 0.3 mM KH2PO4, 2.4 mM CaCl2, 1.3 mM MgSO4, 0.8 mM NaH2PO4, and 10 mM glucose. In the recording chamber the slices were continuously perfused with 35 °C oxygen-bubbled recording saline. In amphetamine (d-amphetamine hemi-sulfate, Sigma-Aldrich) experiments the drug was added to the perfusate (5 uM) after a stable baseline of stimulated transient peaks was reached. Fast-scan cyclic voltammetry recordings were performed with cylindrical 5 μm carbon fiber electrodes positioned at the dorsal striatum ~50 μm below the slice surface. Striatal slices were electrically stimulated using a bipolar stainless steel electrode placed at a distance of ~100 μm from the recording electrode. Square pulses of 0.2 sec duration were produced by DS3 stimulator (Digitimer Ltd, Hertfordshire, UK) that was triggered by a Master-8 pulse generator (A.M.P.I., Israel). Stimulus magnitude was selected by plotting a current–response curve to single pulse stimulations and selecting the minimum value that reliably produced the maximal response. Triangular voltage ramps from −450 mV holding potential to +900 mV over 9 ms (scan rate of 300 mV/ms) were applied to the carbon fiber electrode at 100 ms intervals. Current was recorded with an Axopatch 200B amplifier (Molecular Devices LLC, CA, USA) filtered with 5 kHz low-pass Bessel filter and digitized at 40 kHz (ITC-18 board, InstruTech, NY, USA). Triangular wave generation and data acquisition were controlled and the recorded transients were characterized by a computer routine in IGOR Pro (WaveMetrics Inc, OR, USA) ^4,5^. Background-subtracted cyclic voltammograms, obtained with 1 uM dopamine solution (dopamine-HCl, Sigma-Aldrich), were used to calibrate the electrodes. The DA terminals were stimulated either with single electrical pulses at 2 min intervals or by a burst stimulation of 5 pulses at 20 Hz, to study the release probability of the terminals.

Measurement of dopamine release in vivo by microdialysis

Mice were anaesthetized with isoflurane (5% induction, 1-2% maintenance, Virbac, Carros, France) mixed with oxygen (flow rate 0.8-1.0 L/min), placed in a stereotactic frame (David Kopf Instruments, Tujunga, CA, USA), the skull exposed via a small incision, and a small unilateral craniotomy was performed to allow the placement of the microdialysis guide cannula. The guide cannula (MAB 4.9.1C, AgnTho’s, Lidingö, Sweden) was lowered to a coordinate aimed at the NAcc (anteroposterior +1.30 mm in relation to bregma, laterally +/- 0.70 mm in relation to midline and at –x.xx mm from the skull level) and at the dorsal striatum (anteroposterior +0.60 mm in relation to bregma, laterally +/- 1.20 mm in relation to midline and at –2.80 mm from the skull level), according to Paxinos and Franklin (2001). The guide cannula was secured to the skull with two stainless steel screws and dental cement, and a stainless steel obturators were inserted to keep patency at the period of recovery. For postoperative care mice received carprofen (5 mg/kg. s.c., Norbrook, Newry, UK) and buprenorphin (0.1 mg/kg, s.c., Indivior, Slough, UK) and a recovery period of 1 week was attained before commencing in vivo microdialysis testing. The in vivo microdialysis experiment was started by removing the stainless steel obturator and inserting microdialysis probe (MAB 4.9.1Cu, 1.0 mm membrane, o.d 0.24 mm) into the guide cannula. A constant flow (2 μl/min) with modified Ringer solution (147 mM NaCl, 1.2 mM CaCl2, 2.7 mM KCl, 1.0 mM MgCl2, and 0.04 mM ascorbic acid) was kept using a microsyringe pump (Hamilton, USA). After stabilization of 2 h, amphetamine (dissolved in saline) was injected at 3 mg/kg, i.p. The microdialysis samples (every 20 min, 40 μl/sample) were collected over 4 hours. The concentration of dopamine was measured with high-performance liquid chromatography (HPLC) as described previously ^6^.

**References**

1. Endo, T. *et al.* Automated test of behavioral flexibility in mice using a behavioral sequencing task in IntelliCage. *Behav Brain Res* **221**, 172-81 (2011).

2. Kitanaka, J. *et al.* Pretreatment with nomifensine or nomifensine analogue 4-phenyl-1,2,3,4-tetrahydroisoquinoline augments methamphetamine-induced stereotypical behavior in mice. *Brain Res* **1439**, 15-26 (2012).

3. Lahti, L. *et al.* Differentiation and molecular heterogeneity of inhibitory and excitatory neurons associated with midbrain dopaminergic nuclei. *Development* **143**, 516-29 (2016).

4. Mosharov, E.V. & Sulzer, D. Analysis of exocytotic events recorded by amperometry. *Nat Methods* **2**, 651-8 (2005).

5. Mosharov, E.V. Analysis of single-vesicle exocytotic events recorded by amperometry. *Methods Mol Biol* **440**, 315-27 (2008).

6. Kopra, J. *et al.* Dampened Amphetamine-Stimulated Behavior and Altered Dopamine Transporter Function in the Absence of Brain GDNF. *J Neurosci* **37**, 1581-1590 (2017).
